# Initial organizational practices and contextual factors related to brain injury screening and referral in intimate partner violence serving community- based organizations

**DOI:** 10.21203/rs.3.rs-10071127/v1

**Published:** 2026-07-31

**Authors:** Emiliane L. Pereira, Paul A. Estabrooks, Thet W. Nwe, Peggy Reisher, Kathy S. Chiou, Christopher Wichman, Shireen S. Rajaram

**Affiliations:** University of Nebraska Medical Center; University of Utah; University of Nebraska Medical Center; Brain Injury Association of Nebraska; University of Nebraska- Lincoln; University of Nebraska Medical Center; University of Nebraska Medical Center

**Keywords:** Brain injury, Brain injury screening, Brain injury referral, Intimate partner violence (IPV), Community-based organizations (CBOs), Implementation science, PRISM framework

## Abstract

**Background:**

Early detection of brain injury (BI) among survivors of intimate partner violence (IPV) through screening and referral to follow-up care can help mitigate the impact of IPV-related BI. Despite the high risk for BI among IPV-survivors, BI screening is not standard practice in IPV-serving community-based organizations (CBOs). This project evaluates implementation strategies co-developed with CBOs to embed BI screening and referral processes into their workflows. This paper presents baseline findings from an ongoing stepped-wedge, mixed methods study designed to improve the integration of BI screening and referral practices into IPV-serving CBO.

**Methods:**

Guided by the Practical Robust Implementation and Sustainability Model (PRISM) and the Reach, Effectiveness, Adoption, Implementation, and Maintenance (RE-AIM) framework, baseline data were collected from 12 IPV-serving CBOs in rural and urban areas of a Midwestern state prior to the start of an action learning collaborative (ALC) implementation strategy. CBOs were randomly assigned to three virtual ALC groups of four CBOs each, phased in at four-month intervals. ALCs worked collaboratively, to identify, adapt, and implement strategies to support BI screening and referral. Data sources included organizational records on screening and referral practices, and staff surveys that assessed perceptions of implementation readiness, barriers, and facilitators. The broader parent study uses a participatory, community-engaged, dissemination and implementation research (CEDI) approach across project activities.

**Results:**

Twelve participating CBOs served average monthly from 6 to 340 clients and represented diverse IPV service settings including shelter and walk-in services. At baseline, 7 of the 12 CBOs had conducted at least one BI screening, while only one had completed referrals. Staff survey data highlighted several contextual barriers to implementation, including competing client demands, limited staff readiness to conduct screening and referral, complexity in the BI screening tool, and communication of results to clients. Staff reported strengths including awareness of BI, perceived importance of screening and referral, and management support.

**Conclusion:**

Findings suggest that IPV-serving CBOs are promising but inconsistently prepared for BI screening and referral. The identified practice gaps and contextual factors can guide site-tailored strategies for future efforts to adapt and integrate BI screening and referral into IPV service settings.

## Background

Intimate partner violence (IPV) is a serious yet preventable public health issue that affects millions of people in the U.S. and worldwide (1, 2). IPV refers to violence or aggression that occurs within a close relationship, including a current or former spouse, dating partner, or sexual partner, and can include physical, sexual, or psychological abuse (3). Women report experiencing a wider range of IPV types than men, and they also tend to experience it more severely and repeatedly (4). In the United States, 42% of women report experiencing IPV at some point in their lives (5).

A particularly concerning consequence of IPV-related violence is brain injury (BI). IPV survivors are at high risk for injuries to the head, neck, and face, as well as strangulation, which can result in BI(6–9).Approximately 50%–90% of women who experience IPV have sustained a BI, with many experiencing repeated injuries (10–12). These injuries can have long-lasting consequences for cognitive, psychosocial, and physical functioning(7, 13–16). This includes memory loss, fatigue, depression, confusion, dizziness, impaired judgement, and challenges with daily function (13, 17).

Unfortunately, IPV-related BI is often overlooked and under-detected for many reasons. IPV survivors may not seek medical care due to stigma, lack of awareness of BI and its impact, safety concerns, and lack of autonomy or access to care. BI is not routinely assessed in many service settings. There is an overlap between BI symptoms and common IPV consequences such as post-traumatic stress. The symptoms of IPV-related BI may not be immediately visible or obvious (18), further complicating diagnosis. Finally, statistics on IPV-related injuries, including BIs, reflect only those who seek services or support, leaving a significant portion of cases unaccounted for (2). As a result, estimates of IPV-related BI prevalence rates range from 1 in 5 to as high as 5 in 5 people (10, 19, 20). Despite the high risk of BI among IPV survivors, little is known about the extent to which IPV-serving community-based organizations (CBOs) have incorporated BI screening and referral into routine practice or about the contextual factors that may influence implementation in these settings.

Among the many interest-holders involved in coordinated community responses to IPV secondary prevention, IPV-serving CBOs play a particularly important role in providing survivors with supportive services such as shelter, legal assistance, food security, advocacy, and social support (21). Women survivors are more likely than men to seek services from CBOs, particularly when IPV is severe or repeated (22). These CBOs may be especially important for immigrant, racial/ethnic minority, and other marginalized survivors who often face systemic barriers when seeking help (23, 24). However, due to the complexity of their clients’ needs, inadequate awareness of IPV-related BI, and insufficient training and resources to identify and appropriately respond, BI screening and referral are not yet standard practice among IPV-serving CBOs (25, 26). Although prior work suggests that BI screening should be integrated within trauma-informed service delivery systems (2) (limited evidence exists on the most effective strategies to incorporate BI screening and referral services within IPV-serving CBO workflows (27–29). Therefore, our study was designed to examine implementation strategies for the secondary prevention of IPV-related BI to guide future policies for early screening and referral, ultimately reducing the neurological consequences of BI in this population. We hypothesized that dissemination and implementation strategies derived from CBOs and other interest-holders, and informed by day-to-day interactions with IPV survivors, could improve timely screening and referral of IPV survivors who are at risk for a BI.

The overall aim of the parent project is to evaluate implementation strategies co-developed with IPV-serving CBOs to integrate BI screening and referral processes into their workflows. A participatory and bundled, cross-organizational strategy, the Action Learning Collaborative (ALC), was used to support contextual assessment, co-develop strategies, and share experiences with implementation over time (30). However, the purpose of this paper is to present baseline findings on CBO screening and referral practices and contextual factors collected prior to the beginning of each ALC cohort. By characterizing the organizational starting point before the ALC collaborative implementation support began, this paper identifies current practice gaps, CBO strengths, and potential directions for co-designed site specific improvement (31). The findings from the initial phase of this study can inform the integration of BI screening and referral strategies into CBO workflows. This can provide evidence to guide at-risk populations, service providers, policymakers, and other interest-holders in advancing sustainable approaches for IPV-related BI screening and referral.

### Implementation Framework

We used RE-AIM (Reach, Effectiveness, Adoption, Implementation and Maintenance) framework to assess key implementation outcomes across CBOs (32). In parallel, we applied the Practical, Robust Implementation and Sustainability Model (PRISM) to assess the contextual factors influencing reach, adoption, implementation, and maintenance (33). These included organizational characteristics, multilevel perceptions of BI screening and referral intervention, external environment, and infrastructure to support implementation and sustainability (34). As this paper focuses on contextual factors, RE-AIM and PRISM are used to organize initial CBO practices that may shape future implementation efforts.

## Methods

This paper reports baseline data from an ongoing mixed-method, stepped-wedge implementation study with IPV-serving CBOs. The broader parent study was designed to support the integration of BI screening and referral processes into CBO workflows using a participatory, community-engaged dissemination and implementation (CEDI) approach (35). This manuscript was prepared in accordance with the Standards for Reporting Implementation Studies (StaRI) reporting guidelines (36).

### Design

**Figure 1:**
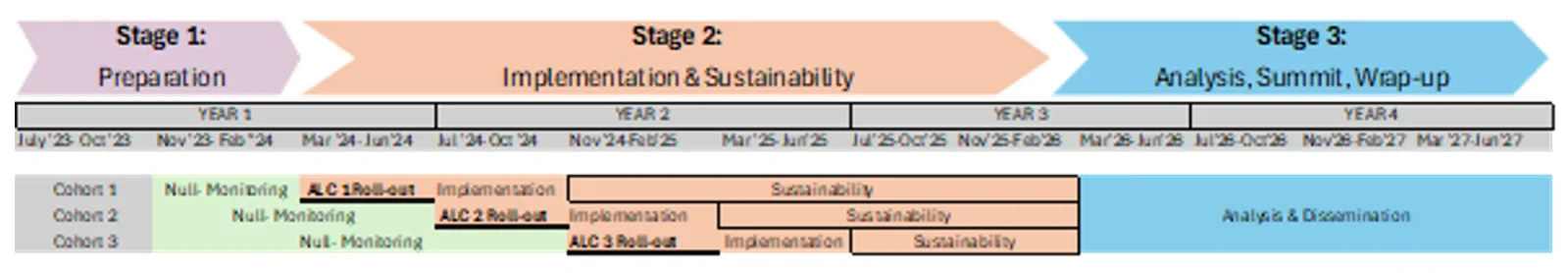
Stepped- wedge implementation of brain Injury across three ALC cohorts

We employed a stepped-wedge design to introduce implementation support activities using participatory ACLs. Twelve CBOs were randomly assigned to three ALC cohorts, with four CBOs per cohort, over a four-year period. The study was organized into three stages: Stage 1 (preparation), Stage 2 (implementation and sustainability), and Stage 3 (analysis and summit) (see Figure 1). In this paper, only baseline data collected prior to the start of each ALC cohort are included.

### Implementation Strategy

To support the adoption, implementation, and maintenance of brain injury (BI) screening and referral practices, the parent study uses a bundled, participatory implementation support approach that combines core project elements with locally tailored strategies. The core elements included: (1) establishment of advisory boards to guide project oversight and sustainability; (2) ALCs to facilitate peer learning and co-selection of strategies; (3) tailored training for CBO staff delivered by the Brain Injury Association; and (4) prior Expert Recommendation for Implementation Change (ERIC)-aligned (30) strategy-prioritization work to inform candidate implementation approaches for each CBO Building on this foundation, each CBO selected and adapted one to two additional strategies to fit their local context and workflows (37). These tailored strategies were refined through ongoing ALC meetings.

### Aims

The purpose of this paper is to present baseline findings on BI screening and referral practices and contextual factors in IPV-serving CBOs. Specifically, we report organizational screening and referral activity and staff survey data collected prior to the beginning of each ALC cohort to characterize the organizational starting point before the collaborative implementation support began.

### Setting and Characteristics of Sites and Participants

Twelve CBOs located in rural and urban areas of a Midwestern state participated in the study. The CBOs were randomized into three ALC cohorts with four CBOs per cohort. Participating sites varied by type (shelter and non-shelter) and organizational size, serving an average of 83 clients per month (range: 6-340). The majority of clients served were women (90%), most of whom were between 25 and 59 years of age (see Table 1). Staff participants included direct service providers, supervisors, administrative leaders, training staff, quality assurance personnel, and others involved in service delivery and organizational operations.

### Data Collection

The parent study uses a mixed-method design and includes quantitative and qualitative data from multiple sources, including staff surveys (at baseline, 8 months, and 20 months), focus groups (two time points), observations from ALC meetings, and virtual interviews with IPV survivors. Outcome measures across the parent study were guided by RE-AIM and PRISM frameworks. For the purpose of his paper, two baseline data sources are included. First, organizational records were used to characterize BI screening and referral practices in the participating CBOs during the baseline period. Second, baseline staff surveys were used to assess the contextual factors relevant to the implementation of BI screening and referral. Staff items were scored on a 1-5 Likert scale ranging from strongly disagree to strongly agree. The staff survey was developed for this study and is provided in full as Additional file 1. Measures of reach (proportion of clients offered BI screening), effectiveness (proportion of those screened and eligible who completed a BI follow-up referral visit with the Brain Injury Association), and PRISM contextual influences were assessed at baseline and included in this paper. For example, staff surveys were conducted at baseline to capture organizational perspectives using the PRISM framework. The surveys included items across the following domains: organizational perspective, patient perspective, external environment, and implementation and sustainability infrastructure.

Other data collected in the parent study but not included in this paper include organizational-level assessments of adoption (CBO completion of an initial BI screening), implementation (consistent use of screening/referral process), and maintenance (use of screening/referral process 12–20 months after ALC activities).

Tables 2–4 summarize the parent study measures guided by RE-AIM and PRISM, and this paper will include only the baseline organizational screening/referral data and baseline staff survey findings.

### Data analysis

We conducted descriptive analyses to summarize BI screenings and referrals completed during the baseline period (November 2023–February 2024) using CBO monthly reports. For each CBO, the total number of screenings and referrals completed during the baseline was calculated by ALC cohort. Baseline staff survey data were analyzed using descriptive statistics to summarize participant demographics and professional characteristics. Survey items were mapped to the corresponding PRISM framework domains and subdomains. Three research team members (TN, EP, PE) independently coded each survey question. Two team members (TN, PE) reviewed the assignments and indicated agreement or disagreement. Discrepancies were resolved when at least two coders reached consensus. For each survey item, means and standard deviations were calculated to identify strengths and areas for improvement, within and across domains. In combination, these analyses were intended to characterize initial organizational practices rather than evaluate intervention effects.

## Results

### Screening and Referrals

**Figure 2:**
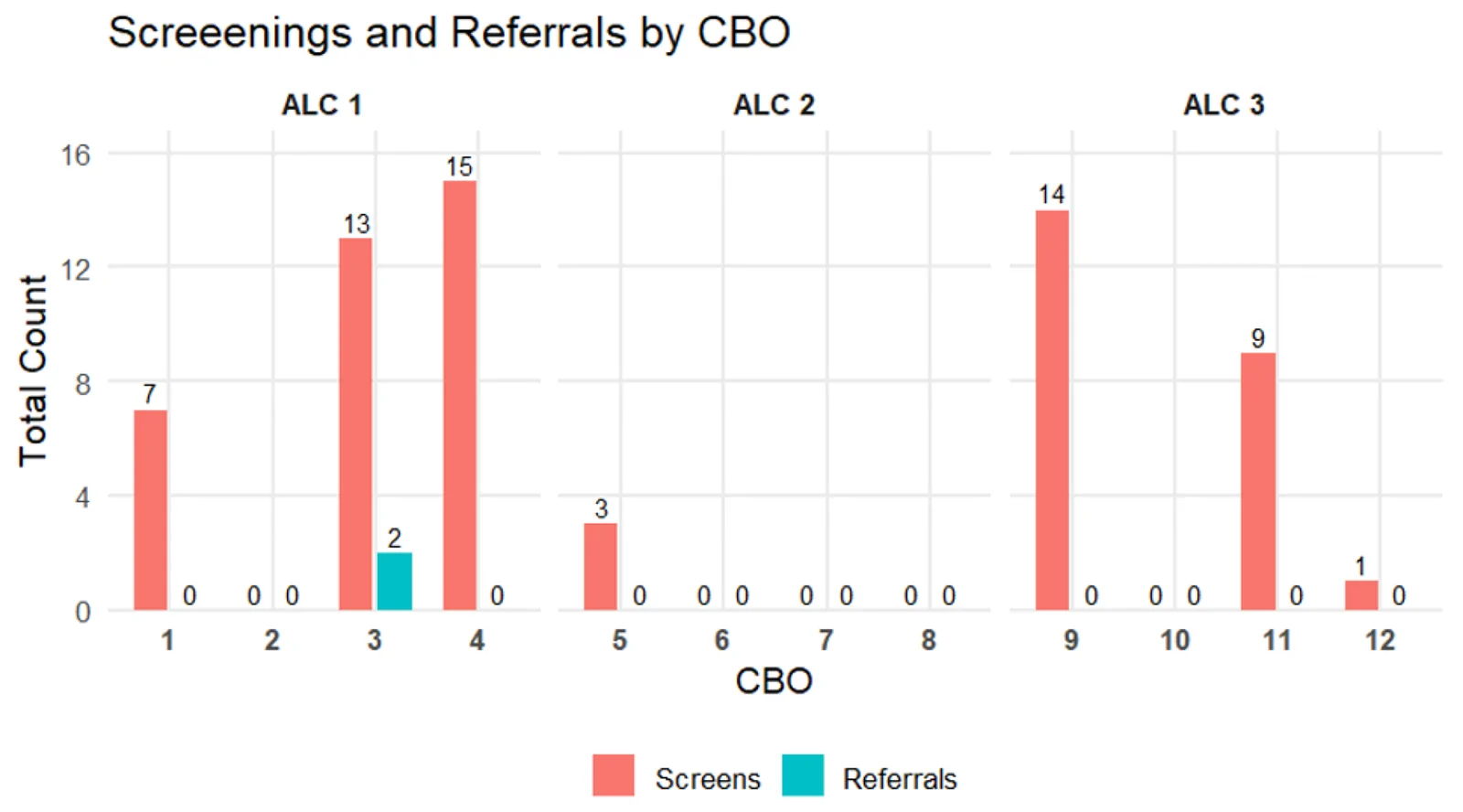
Baseline screenings and referrals

During the baseline phase of the project, before the initiation of ALC meetings, BI screenings and referrals activity varied across CBOs and ALC cohorts. For example, at baseline, in the ALC 1 cohort, 3 out of 4 CBOs completed screenings, and only one CBO completed referrals. In contrast, in the ALC 2 cohort, only 1 out of 4 CBOs completed screenings, with no referrals. The ALC 3 cohort showed a similar pattern to ALC 1, and 3 out of 4 CBOs completed screenings but no referrals. Across all 12 CBOs, 7 completed at least one BI screening at baseline, whereas only 1 completed any referrals. These findings suggest that some CBOs had initiated incorporating BI screening into practice before formal implementation support, but referral pathways were far less established. See Figure 2 for detailed screenings and referrals counts.

### Baseline staff survey

A total of 72 staff surveys were completed. The majority of respondents were female (92%), followed by gender non-conforming participants (4%). Most staff reported having a college degree (38%) and were between 26 and 35 years old (33%). Respondents represented a range of organizational roles and were most commonly client service providers but also included supervisors, training staff, quality assurance personnel, and others involved in organizational operations and service delivery. The majority identified as White (83%). See Table 5 for demographic characteristics of the staff participants.

### PRISM-Guided Analysis of Baseline Staff Survey

Survey items were systematically categorized within PRISM domains and sub-domains, as presented in Table 6. Analysis of staff survey responses using PRISM domain constructs highlighted several contextual strengths and barriers at both the survivor and organizational levels. Because items were scored on a 1-5 Likert scale, lowest mean scores indicated lower implementation-related factors, whereas highest mean scores indicated stronger agreement and more favorable perceptions of implementation factors. The lowest mean score (M = 3.3) was observed in the program intervention, patient perspective domain, specifically regarding the seamlessness of the transition from BI screening to referral. Staff readiness to conduct screenings and referrals was also relatively low (M = 3.4), suggesting that many organizations entered the project without strong confidence in their ability to carry out these processes consistently. Similarly, the ease with which clients could understand the screener results received a low mean score of 3.4. Other lower-scoring items pointed to challenges related to training and support, the presence of a dedicated champion or team, and the ease of following the referral process.

In contrast, items with higher mean scores reflected staff recognition of the complexity of serving this population. For example, advocates acknowledged clients’ competing demands such as housing and childcare (M = 4.5). In addition, most staff reported prior awareness of BI (M = 4.4) and agreed that BI screening and referral is important for their clients (M = 4.3) (see Table 6). Staff also endorsed patient choice, management support, and the availability of community-based follow-up resources, suggesting that participating CBOs entered the project with important implementation assets despite uneven readiness and referral infrastructure.

## Discussion

This paper provides a contextual baseline for understanding how BI screening and referral may be integrated into IPV-serving CBOs. Rather than serving as an early test of strategy effectiveness, these findings clarify the organizational starting point from which future implementation efforts should proceed. In doing so, the paper contributes to identifying the contextual conditions that shape whether, how, and for whom an evidence-based practice can be adopted, implemented, and sustained in real-world settings—a core goal of dissemination and implementation research (38). A central implication of these findings is that screening and referral should not be treated as a single implementation task. Some organizations had already initiated screening before collaborative implementation support began, whereas referral activity was rare. This pattern suggests that establishing a referral pathway may require a different level of organizational coordination, cross-system linkage, staff confidence, and workflow integration than initiating screening alone. This interpretation is consistent with prior work showing that transitions across service settings are often challenging for IPV survivors and that community follow-up pathways can break down even when need is recognized (21). It is also consistent with dissemination and implementation science literature emphasizing that different practice components often encounter different determinants and therefore may require different implementation strategies (39).

The staff survey findings are particularly useful when interpreted using PRISM contextual factors (34). Higher-scoring items suggest that participating CBOs entered the project with important implementation assets, including awareness of BI, recognition of its relevance for clients, management support, and appreciation of the competing demands survivors face. Lower-scoring items point to issues of readiness, communication of screening results, training and support, and the seamlessness of transition from screening to referral. In PRISM terms, these findings suggest that the key challenge is not simply whether BI screening is viewed favorably, but whether there is adequate fit among the intervention, staff capacity, referral processes, and the realities of survivors’ live(33, 40). This interpretation aligns with PRISM’s emphasis on multilevel context and the importance of aligning implementation approaches with organizational characteristics, external resources, and implementation infrastructure(34)

The findings also reinforce a broader lesson from implementation science that describes context as key to implementation success and not just a description of the setting where implementation occurs (38). In this study, the relevant contextual conditions include the complexity of clients’ immediate needs, uneven staff readiness, uncertainty about communicating screening results, and a gap in referral continuity. These are not minor operational details, but rather they are targets for future implementation strategies as both potential moderators of success and mechanisms of change (41). Reviews of implementation frameworks have repeatedly emphasized that attention to context is necessary for explaining implementation variation, while emerging work in community settings has highlighted the importance of readiness-building and locally feasible strategy selection before expecting sustained practice change (42). This also aligns with the growing literature suggesting that screening-related innovations in trauma-exposed populations cannot be implemented successfully through technical training alone (27). In crisis-oriented and trauma-affected service settings, implementation depends on whether the new practice can be integrated into existing trauma-informed workflows without increasing burden, reducing flexibility, or compromising survivor-centered care. Systematic review of evidence on trauma-informed organizational change similarly suggests that successful implementation depends on organizational culture, training, coaching, leadership support, and the ability to respond to competing priorities (43). In that sense, the present findings do not simply document barriers, they identify the contextual constraints that any BI screening and referral strategy must address to be workable in IPV-serving CBOs.

This study adds to the dissemination and implementation literature in two ways. First, it extends the emerging literature on IPV-related BI by shifting attention from problem recognition to implementation context in real-world community settings, where screening and referral are critically needed. Second, we illustrate the value of using initial contextual assessment to inform later strategy selection and tailoring. Peer-reviewed implementation literature increasingly argues that strategy selection should be linked to identified contextual factors rather than applied generically, particularly in community settings where resource constraints, organizational diversity, and workflow variability are pronounced (38, 44). The present findings support that position by showing that readiness, referral continuity, training support, and workflow fit are likely to be central targets for site-tailored improvement efforts in IPV-serving CBOs.

### Limitations of the study

Several limitations should be noted. First, these findings reflect baseline conditions in 12 CBOs from one U.S. state and should be interpreted cautiously in other IPV service contexts. Second, the survey results reflect staff perspectives and do not fully capture survivor experiences of screening, referral, and follow-up. Third, this paper focuses intentionally on initial organizational practices and contextual factors. It does not assess whether the broader ALC implementation support approach improved adoption, implementation, or maintenance over time. At the same time, the inclusion of both rural and urban organizations, as well as shelter and non-shelter settings, is a strength for a contextual baseline analysis because it captures meaningful variation in the environments where implementation will need to occur.

## Conclusion

Taken together, these findings suggest that the key issue in IPV-serving CBOs is not simply whether BI screening is valued but whether organizations have the contextual infrastructure needed for screening to lead to timely referral and follow-up support. That distinction matters for both practice and research. For practice, it points to the need for implementation efforts that are responsive to local workflow, staffing, and referral ecology. For research, it highlights the importance of evaluating how site-tailored strategies address specific determinants over time rather than assuming that one uniform package will be equally workable across organizations.

## Supplementary Material

This is a list of supplementary files associated with this preprint. Click to download.

- AdditionalFile1Staffsurvey.docx

## Figures and Tables

**Table 1.** Study implementation sites

ALC group	Site	Rurality	Clients per site, Mean (SD)
ALC 1	1	Rural	70 (4.4)
ALC 1	2	Urban	15.2 (0.5)
ALC 1	3	Urban	27.7 (3.1)
ALC 1	4	Rural	35 (31.8)
ALC 2	5	Rural	97.5 (5.7)
ALC 2	6	Urban	6.8 (3.5)
ALC 2	7	Rural	38.5 (9.7)
ALC 2	8	Urban	224.5 (5.6)
ALC 3	9	Rural	4.3 (1.5)
ALC 3	10	Rural	10.5 (3.9)
ALC 3	11	Urban	19 (2.2)
ALC 3	12	Urban	340 (91.2)

**Table 2.** Aim 1- Brain injury intervention guided by RE-AIM/PRISM framework

RE-AIM Dimension	Measures	Data sources(s)	Data type	Level
Adoption Absolute number, proportion of CBOs that completed an initial brain injury screening with an IPV survivor	% of screeners completed by CBOs Barriers and facilitators to adoption	Monthly reports Redcap screener tool survey Observation Staff survey Focus groups	Quant Quali	Organizational

Implementation Ongoing use of the brain injury and referral process based on consisted screening over the initial implementation period	% of CBOs site completing ≥1 BI screening during the implementation period Documented adaptations to the screener tool Barriers & facilitators to implementation	Monthly reports Redcap screener tool survey Adaptation tracking	Quant Quali	Organizational Client

Maintenance- Ongoing use of brain injury screening and referral process during sustainability period (12 to 20 months post ALC activities)	% of CBOs site completing ≥1 BI screening during the sustainability period Barriers & facilitators to sustained use	Monthly reports Redcap screener tool survey Focus groups	Quant Quali	Organizational Client

**Table 3.** Aim 2- Brain injury intervention guided by RE-AIM/PRISM framework

RE-AIM Dimension	Measures	Data sources(s)	Data type	Level
Reach The proportion of the total number of clients each CBO serves in a specific period who were offered the BI screener for a probable BI	Number of clients completed BI screen ÷ total clients seen by the CBO in the same period	Monthly reports Redcap screener tool survey	Quant	Client

Effectiveness The proportion of clients who are screened and have a probable BI that are referred to and completed a visit (in person or online) with a BIA advocate	Number of clients with probable BI completing a BIA visit ÷ number of clients screened with a probable BI result in the same period	Quarterly reports	Quant	Client

**Table 4.** PRISM Contextual Factors Measures Assessed

PRISM Constructs	Measures Assessed	Data Source(s)	Data Type	Level
	Program Intervention (Organizational Perspective)			
**Program Intervention (Organizational Perspective)**	Readiness; Coordination across departments & specialties; Usability & adaptability	Staff survey; ALC meeting observations	Quant; Quali	Organizational
	Program Intervention (Patient Perspective)			
**Program Intervention (Patient Perspective)**	Patient centeredness; Provides patient choice; Addresses patient barriers; Burden (complexity & cost); Seamlessness of transition between program elements	Staff survey; Survivors’ interviews; ALC meeting observations	Quant; Quali	Client
	External Environment			
**External Environment**	Community resources	Staff survey; ALC meeting observations	Quant; Quali	Organizational
	Implementation & Sustainability Infrastructure			
**Implementation & Sustainability Infrastructure**	Dedicated team; Usability & adaptability	Staff survey; ALC meeting observations	Quant; Quali	Organizational
	Recipients: Organizational Characteristics			
**Recipients: Organizational Characteristics**	Organizational health & culture; Management support & communication; Shared goals & cooperation; Data & decision support; Systems & training; Expectation of sustainability; Knowledge & beliefs; Addresses patient barriers	Staff survey; Monthly report; ALC meeting observations	Quant; Quali	Organizational
	Recipients: IPV Survivors’ Characteristics			
**Recipients: IPV Survivors’ Characteristics**	Demographics; Disease burden; Competing demands	Monthly report; Screening data; Survivors’ interviews	Quant; Quali	Client

**Note.** Quant = Quantitative; Quali = Qualitative

**Table 5.** Demographic Characteristics of Staff Participants

Variable	Category	n	Percent
Age	26-35 years	24	33.3%
	19-25 years	18	25.0%
	46-55 years	15	20.8%
	36-45 years	11	15.3%
	Over 55 years	2	2.8%
	Prefer not to answer	2	2.8%
Gender	Female	66	91.7%
	Gender nonconforming	3	4.2%
	Prefer not to answer	2	2.8%
	Male	1	1.4%
Education	College degree	27	37.5%
	Some college	15	20.8%
	Post-graduate (Masters and above)	14	19.4%
	High school/GED	9	12.5%
	Associate degree	5	6.9%
	Prefer not to answer	2	2.8%
Ethnicity	not Hispanic/ Latinx	53	73.6%
	Hispanic/ Latinx	15	20.8%
	Prefer not to answer	4	5.6%
Race	White- Caucasian	60	83.3%
	Prefer not to answer	6	8.3%
	Mixed-racial background	4	5.6%
	African American	1	1.4%
	Native American	1	1.4%
Role	Client service provider	47	65.3%
	Supervise administrative staff	10	13.9%
	Supervise other managers	7	9.7%
	Quality assurance	3	4.2%
	Training staff	6	8.3%
	Other	20	27.8%

**Note.** Percentages for role variable do not sum to 100% because respondents could select multiple categories

**Table 6.** Baseline staff survey results

Survey Question	PRISM Domain	PRISM Sub-domain	Mean	SD
Advocates recognize that clients may have competing demands such as housing, childcare, etc.	Program Intervention (Patient Perspective)	Burden (complexity and cost)	4.5	0.8
I have heard about brain injury screening and referral before taking this survey.	Recipients (Organizational Characteristics)	Knowledge and beliefs	4.4	1.1
Brain injury screening and referral is important for our clients.	Program Intervention (Patient Perspective)	Patient centeredness	4.3	0.9
Clients are provided with the choice not to participate in the brain injury screening.	Program Intervention (Patient Perspective)	Provides patient choice	4.3	0.9
Advocates are aware of the barriers to clients’ participation in the brain injury screening and referral process.	Recipients (Organizational Characteristics)	Addresses patient barriers	4.3	0.7
Brain injury screening and referral can be embedded into the workflow of the organization.	Implementation & Sustainability Infrastructure	Usability and adaptability	4	0.8
Direct service staff have the support of management for the brain injury screening program.	Recipients (Organizational Characteristics)	Management support and communication	4	0.8
There are community services such as the Brain Injury Alliance of Nebraska that can provide follow-up support for the brain injury screening program.	External Environment	Community resources	4	0.8
The brain injury screening program has received support from key managers in the organization.	Recipients (Organizational Characteristics)	Management support and communication	4	0.8
Cross-department coordination is needed in screening and referral of a brain injury.	Program Intervention (Organizational Perspective)	Coordination across departments and specialties	3.9	0.9
The brain injury screener can be easily used by staff.	Program Intervention (Organizational Perspective)	Usability and adaptability	3.8	0.9
The brain screening and referral process is responsive to the concerns of clients.	Program Intervention (Patient Perspective)	Patient centeredness	3.8	0.8
The organization has shared goals pertaining to the brain injury screening program.	Recipients (Organizational Characteristics)	Shared goals and cooperation	3.8	0.9
There are systems in place to report the results of the brain injury screening program to the research team.	Recipients (Organizational Characteristics)	Data and decision support	3.8	0.8
The results of the brain injury screener are immediately shared with the client.	Program Intervention (Patient Perspective)	Patient centeredness	3.7	0.9
The organization has the necessary financial capacity to implement the brain injury screening program.	Recipients (Organizational Characteristics)	Organizational health and culture	3.7	0.8
Key staff members expect the brain injury screening program to be sustainable.	Recipients (Organizational Characteristics)	Expectation of sustainability	3.7	0.7
Clients can access support services in the community to follow-up on the results of the brain injury screening program.	External Environment	Community resources	3.7	0.8
The questions in the brain injury screener are easy for clients to understand.	Program Intervention (Patient Perspective)	Patient centeredness	3.6	0.9
The brain injury screener is safe for the mental health of clients.	Program Intervention (Patient Perspective)	Patient centeredness	3.5	0.7
The brain injury referral process is easy for clients to follow.	Program Intervention (Patient Perspective)	Burden (complexity and cost)	3.5	0.8
Staff receive adequate training and support for the brain injury screening program.	Recipients (Organizational Characteristics)	Systems and training	3.5	0.9
My organization has a dedicated ‘champion’ or team in charge of the brain injury screening program.	Implementation & Sustainability Infrastructure	Dedicated team	3.5	1
Key staff members are ready to conduct brain injury screening and referral.	Program Intervention (Organizational Perspective)	Readiness	3.4	1.1
The results of the brain injury screener are easy for clients to understand.	Program Intervention (Patient Perspective)	Burden (complexity and cost)	3.4	0.9
For clients, there is a seamless transition from the brain injury screening to the referral process.	Program Intervention (Patient Perspective)	Seamlessness of transition between program elements	3.3	0.9

## Data Availability

The datasets used and/or analyzed during the current study are available from the corresponding author on reasonable request.

## Abbreviations

BI: Brain Injury
CAB: Community-campus advisory board
CBOs: Community-based organizations
ERIC: Expert Recommendations for Implementing Change
IPV: Intimate partner violence
TBI: Traumatic Brain Injury

## References

[1] Mazza M, Marano G, Del Castillo AG, Chieffo D, Monti L, Janiri D, Intimate partner violence: A loop of abuse, depression and victimization. World J Psychiatry. 2021;11(6):215–21. 34168968 10.5498/wjp.v11.i6.215 PMC8209536

[2] Clarke ADA, Copas C, Hannon O, Padgett C, Knight JM, Falkenberg A, Detecting a hidden pandemic: The current state and future direction of screening and assessment tools for intimate partner violence-related brain injury. Neurosci Biobehav Rev. 2024;167:105912. 39368636 10.1016/j.neubiorev.2024.105912

[3] Centers for Disease Control and Prevention. About intimate partner violence: Centers for Disease Control and Prevention; 2026 [Available from: https://www.cdc.gov/intimate-partner-violence/about/index.html.

[4] Fanslow JL, Mellar BM, Gulliver PJ, McIntosh TKD. Evidence of Gender Asymmetry in Intimate Partner Violence Experience at the Population-Level. Journal of Interpersonal Violence. 2023;38(15-16):9159–88. 37032556 10.1177/08862605231163646 PMC10668541

[5] Sharon G. Smith SK Richardson LaTonia, Basile Kathleen C., , Norah W Friar JC, Kudon Hui Zhang, and Leemi Ruth W.. The National Intimate Partner and Sexual Violence Survey: 2016/2017 State Report. . Atlanta (GA): Centers for Disease Control and Prevention, National Center for Injury Prevention and Control; 2023.

[6] Liu LY, Bush WS, Koyuturk M, Karakurt G. Interplay between traumatic brain injury and intimate partner violence: data driven analysis utilizing electronic health records. BMC Womens Health. 2020;20(1):269. 33287806 10.1186/s12905-020-01104-4 PMC7720451

[7] Valera EM, Daugherty JC, Scott OC, Berenbaum H. Strangulation as an Acquired Brain Injury in Intimate–Partner Violence and Its Relationship to Cognitive and Psychological Functioning: A Preliminary Study. Journal of Head Trauma Rehabilitation. 2022;37(1):15–23. 34985030 10.1097/HTR.0000000000000755 PMC8882437

[8] Karr JE, White AE, Logan TK. Depression, anxiety, and posttraumatic stress in women with and without brain injuries due to intimate partner violence: Psychometric evaluation of measurement approaches and group comparisons. Rehabilitation psychology. 2025;70(2):170–81. 39172371 10.1037/rep0000570 PMC12004542

[9] Rajaram SS, Reisher P, Garlinghouse M, Chiou KS, Higgins KD, New-Aaron M, Intimate Partner Violence and Brain Injury Screening. Violence Against Women. 2021;27(10):1548–65. 32838674 10.1177/1077801220947164

[10] Esopenko C, Jain D, Adhikari SP, Dams-O’Connor K, Ellis M, Haag HL, Intimate Partner Violence-Related Brain Injury: Unmasking and Addressing the Gaps. J Neurotrauma. 2024;41(19-20):2219–37. 38323539 10.1089/neu.2023.0543 PMC11564844

[11] Haag HL, Jones D, Joseph T, Colantonio A. Battered and Brain Injured: Traumatic Brain Injury Among Women Survivors of Intimate Partner Violence—A Scoping Review. Trauma, Violence, & Abuse. 2022;23(4):1270–87. 10.1177/1524838019850623 PMC9425721 31170896

[12] Campbell JK, Joseph A-LC, Rothman EF, Valera EM. The Prevalence of Brain Injury Among Survivors and Perpetrators of Intimate Partner Violence and the Prevalence of Violence Victimization and Perpetration Among People With Brain Injury: a Scoping Review. Current Epidemiology Reports. 2022;9(4):290–315.

[13] Jenkins ND, Ritchie CW, Ritchie K, Terrera GM, Stewart WA-O. Intimate partner violence, traumatic brain injury and long-term mental health outcomes in midlife: the Drake IPV study. LID - 10.1136/bmjment-2024-301439 [doi] LID - e301439. 2025(2755-9734 (Electronic)). PMC12161325 40490274

[14] Toccalino D, Moore A, Cripps E, Gutierrez SC, Colantonio A, Wickens CM, Exploring the intersection of brain injury and mental health in survivors of intimate partner violence: A scoping review. 2023(2296-2565 (Electronic)). 10.3389/fpubh.2023.1100549 PMC10018197 36935693

[15] Makovec Knight J, Hannon O, Spitz G, Astridge A, Copas C, Duarte Martins B, Brain Injury Within Intimate Partner Violence: What Are the Cognitive Effects? J Neurotrauma. 2026;43(3-4):269–79. 41164911 10.1177/08977151251388070

[16] Hughes NC, Osama TA, Rigney GH, Jo J, Williams K, Zuckerman SL, Head injuries in intimate partner violence: cognitive, motor, and psychiatric symptoms. 2026(1362-301X (Electronic)). 10.1080/02699052.2026.2628052 41674060

[17] National Academies of Sciences E, and Medicine,. Health Conditions Related to Intimate Partner Violence. In: Bell C, Curry S, editors. Essential Health Care Services Addressing Intimate Partner Violence. Washington (DC): National Academies Press; 2024. 39078927

[18] Stiller M, Wilson ML, Barnighausen T, Adedimeji A, Lewis E, Abio A. Help-seeking behaviors among survivors of intimate partner violence during pregnancy in 54 low- and middle-income countries: evidence from Demographic and Health Survey data. BMC Public Health. 2025;25(1):413. 39893365 10.1186/s12889-025-21421-3 PMC11787739

[19] Callaway CCM, Ch’Ang JH. Traumatic Brain Injury in Intimate Partner Violence. Current Pain and Headache Reports. 2025;29(1). 10.1007/s11916-024-01324-5 39754618

[20] Sardinha L, Maheu-Giroux M, Stöckl H, Meyer SR, García-Moreno C. Global, regional, and national prevalence estimates of physical or sexual, or both, intimate partner violence against women in 2018. Lancet. 2022;399(10327):803–13. 35182472 10.1016/S0140-6736(21)02664-7 PMC8885817

[21] Evans DP, Pawcio J, Wyckoff K, Wilkers L. “And then the person sort of just drops off the radar‖”: barriers in the transition from hospital to community-based care among survivors of intimate partner violence in Metropolitan Atlanta. Front Public Health. 2024;12:1332779. 38841664 10.3389/fpubh.2024.1332779 PMC11150547

[22] PettyJohn ME, Baumler E, Backes B, Brashear B, Temple JR, Wood L. Utilization of services at community-based intimate partner violence agencies: associations with sociodemographic and victimization factors. Journal of Family Violence. 2025;40(5):935–47.

[23] Cho H, Kim W, Gillis K, Goetz K. Factors for Perceived Helpfulness of Support Sources Among Survivors of Intimate Partner Violence. Behavioral Sciences. 2025;15(10):1350. 41153140 10.3390/bs15101350 PMC12561027

[24] Hulley J, Bailey L, Kirkman G, Gibbs GR, Gomersall T, Latif A, Intimate Partner Violence and Barriers to Help-Seeking Among Black, Asian, Minority Ethnic and Immigrant Women: A Qualitative Metasynthesis of Global Research. Trauma, Violence, & Abuse. 2023;24(2):1001–15. 10.1177/15248380211050590 PMC10012394 35107333

[25] Gbadebo A. The Intersection of Intimate Partner Violence, Strangulation, and Brain Injury Screening: A Pilot Project. J Forensic Nurs. 2025;21(1):3–11. 39102334 10.1097/JFN.0000000000000506

[26] Sanhueza-Morales T, Michaelsen S, Touati N, Barrio LRD. Barriers in accessing intimate partner violence services: Intersecting views of immigrant and minority ethnic survivors and community organization workers. Womens Health (Lond). 2025;21:17455057251323091. 40132146 10.1177/17455057251323091 PMC11946282

[27] Hyzak KA, Bunger AC, Bogner J, Davis AK, Corrigan JD. Implementing traumatic brain injury screening in behavioral health treatment settings: results of an explanatory sequential mixed-methods investigation. Implementation Science. 2023;18(1):35. 37587532 10.1186/s13012-023-01289-w PMC10428542

[28] Smart CM, Macoun SJ, Qualls LK, Ellis HA, Baker K, Becker E, Using the Delphi method to develop trauma-informed practice guidelines for neurorehabilitation. Rehabil Psychol. 2025. 10.1037/rep0000615 40354247

[29] Zangi M, Pickering JW, Theadom A, Than M, Snell DL. Mild and moderate traumatic brain injury: Screening, documentation, and referral to concussion services. Australasian Emergency Care. 2025;28(3):213–20. 40204551 10.1016/j.auec.2025.03.007

[30] Powell BJ, Waltz TJ, Chinman MJ, Damschroder LJ, Smith JL, Matthieu MM, A refined compilation of implementation strategies: results from the Expert Recommendations for Implementing Change (ERIC) project. Implementation Science. 2015;10(1):21. 25889199 10.1186/s13012-015-0209-1 PMC4328074

[31] Rajaram SS, Pereira EL, Chiou K, Reisher P, Wichman C, Miller MN, A Delphi Study to prioritize implementation strategies to increase the adoption of screening and referral for brain injury among survivors of intimate partner violence and sexual assault within community-based organizations. LID - rs.3.rs-6925920 [pii] LID - 10.21203/rs.3.rs-6925920/v1 [doi]. 2025(2693-5015 (Electronic)). PMC12911198 41580710

[32] Glasgow RE, Harden SM, Gaglio B, Rabin B, Smith ML, Porter GC, RE-AIM Planning and Evaluation Framework: Adapting to New Science and Practice With a 20-Year Review. Front Public Health. 2019;7:64. 30984733 10.3389/fpubh.2019.00064 PMC6450067

[33] Rabin BA, Cakici J, Golden CA, Estabrooks PA, Glasgow RE, Gaglio B. A citation analysis and scoping systematic review of the operationalization of the Practical, Robust Implementation and Sustainability Model (PRISM). Implement Sci. 2022;17(1):62. 36153628 10.1186/s13012-022-01234-3 PMC9509575

[34] Feldstein AC, Glasgow RE. A Practical, Robust Implementation and Sustainability Model (PRISM) for Integrating Research Findings into Practice. The Joint Commission Journal on Quality and Patient Safety. 2008;34(4):228–43. 18468362 10.1016/s1553-7250(08)34030-6

[35] Shea CM, Young TL, Powell BJ, Rohweder C, Enga ZK, Scott JE, Researcher readiness for participating in community-engaged dissemination and implementation research: a conceptual framework of core competencies. Translational Behavioral Medicine. 2017;7(3):393–404. 28341897 10.1007/s13142-017-0486-0 PMC5645278

[36] Pinnock H, Barwick M, Carpenter CR, Eldridge S, Grandes G, Griffiths CJ. Standards for Reporting Implementation Studies (StaRI) Statement. BMJ. 2017;356:i6795. 28264797 10.1136/bmj.i6795 PMC5421438

[37] Rajaram SS, Pereira EL, Chiou KS, Reisher P, Wichman C, Miller MN, A Delphi study to prioritize implementation strategies to increase the adoption of screening and referral for brain injury among survivors of intimate partner violence and sexual assault within community-based organizations. BMC Public Health. 2026;26(1):642. 41580710 10.1186/s12889-025-25638-0 PMC12911198

[38] Nilsen P, Bernhardsson S. Context matters in implementation science: a scoping review of determinant frameworks that describe contextual determinants for implementation outcomes. BMC Health Serv Res. 2019;19(1):189. 30909897 10.1186/s12913-019-4015-3 PMC6432749

[39] Balis LE, Houghtaling B, Harden SM. Using implementation strategies in community settings: an introduction to the Expert Recommendations for Implementing Change (ERIC) compilation and future directions. Translational Behavioral Medicine. 2022;12(10):965–78. 36039843 10.1093/tbm/ibac061

[40] Jolles MP, Fort MP, Glasgow RE. Aligning the planning, development, and implementation of complex interventions to local contexts with an equity focus: application of the PRISM/RE-AIM Framework. Int J Equity Health. 2024;23(1):41. 38408990 10.1186/s12939-024-02130-6 PMC10898074

[41] Lewis CC, Klasnja P, Lyon AR, Powell BJ, Lengnick-Hall R, Buchanan G, The mechanics of implementation strategies and measures: advancing the study of implementation mechanisms. Implement Sci Commun. 2022;3(1):114. 36273224 10.1186/s43058-022-00358-3 PMC9588220

[42] Hassett L, Moseley AM, Nettlefold L, Pearce LMN, Franke T, Macdonald HM, Building readiness in community-based organisations to enable the implementation of public health interventions for adults and older adults: a scoping review. BMC Public Health. 2025;26(1):2. 41286738 10.1186/s12889-025-25745-y PMC12763873

[43] Goldstein EA-O, Chokshi B, Melendez-Torres GJ, Rios A, Jelley MA-O, Lewis-O’Connor AA-O. Effectiveness of Trauma-Informed Care Implementation in Health Care Settings: Systematic Review of Reviews and Realist Synthesis. 2024(1552–5775 (Electronic)). 10.7812/TPP/23.127 PMC10940237 38444328

[44] Waltz TJ, Powell BJ, Fernandez ME, Abadie B, Damschroder LJ. Choosing implementation strategies to address contextual barriers: diversity in recommendations and future directions. Implement Sci. 2019;14(1):42. 31036028 10.1186/s13012-019-0892-4 PMC6489173

